# Sampling manholes to home in on SARS-CoV-2 infections

**DOI:** 10.1371/journal.pone.0240007

**Published:** 2020-10-05

**Authors:** Richard C. Larson, Oded Berman, Mehdi Nourinejad

**Affiliations:** 1 Institute for Data, Systems, and Society, Massachusetts Institute of Technology, Cambridge, Massachusetts, United States of America; 2 Rotman School of Management, University of Toronto, Toronto, Canada; 3 Department of Civil Engineering, York University, Toronto, Canada; BronxCare Health System, Icahn School of Medicine at Mount Sinai, NY, UNITED STATES

## Abstract

About 50% of individuals infected with the novel Coronavirus (SARS-CoV-2) suffer from intestinal infection as well as respiratory infection. They shed virus in their stool. Municipal sewage systems carry the virus and its genetic remnants. These viral traces can be detected in the sewage entering a wastewater treatment plant (WTP). Such virus signals indicate community infections but not locations of the infection within the community. In this paper, we frame and formulate the problem in a way that leads to algorithmic procedures homing in on locations and/or neighborhoods within the community that are most likely to have infections. Our data source is wastewater sampled and real-time tested from selected manholes. Our algorithms dynamically and adaptively develop a sequence of manholes to sample and test. The algorithms are often finished after 5 to 10 manhole samples, meaning that—in the field—the procedure can be carried out within one day. The goal is to provide timely information that will support faster more productive human testing for viral infection and thus reduce community disease spread. Leveraging the tree graph structure of the sewage system, we develop two algorithms, the first designed for a community that is certified at a given time to have zero infections and the second for a community known to have many infections. For the first, we assume that wastewater at the WTP has just revealed traces of SARS-CoV-2, indicating existence of a “Patient Zero” in the community. This first algorithm identifies the city block in which the infected person resides. For the second, we home in on a most infected neighborhood of the community, where a neighborhood is usually several city blocks. We present extensive computational results, some applied to a small New England city.

## 1. Introduction

From its origins in Wuhan, China in late 2019, the novel Coronavirus (SARS-CoV-2) has spread quickly around the world, creating a disease in humans—COVID-19—that has infected millions and killed hundreds of thousands. The virus has many unusual properties that are still be researched and discovered. One distressing property is that 40 to perhaps 80 percent of infected and virus-shedding individuals are asymptomatic [1, 2]. Another is a relatively long incubation time from becoming infected to showing physical symptoms, up to 14 days with a median time of about 5 days [3].

### 1.1. SARS-CoV-2 in human wastewater

We focus on a third unusual property: SARS-CoV-2 not only attacks human lungs, but it can also reside and present symptoms in other parts of the body, including the human digestive track and particularly the intestines [4]. As a result, the SARS-CoV-2 virus and/or related genetic remnants (viral RNA—ribonucleic acid) often appear in the fecal matter of COVID-19 patients. These genetic materials, excreted in stool, positively affirm that the patient is infected with COVID-19. About 50 per cent of COVID-19 patients present such in-stool markers [5, 6].

Since human waste is flushed down the toilet, any included genetic markers and/or chemicals are detectable in raw sewage. Much of the health condition of the human body can be determined by analyses of sewage, leveraging an emerging field called “Wastewater-Based Epidemiology” (WBE) [7]. Recent research [8] has asserted that detailed analysis of a community’s sewage flowing into its sewage treatment plant (STP) can now theoretically detect the presence of virus remnants emanating from just one infected person in a contributing population of up to 2,000,000. The potential sensitivity of large city sewage testing is remarkably high.

Testing for COVID-19 is vital for adjusting public policies related to its control. But testing each human in a population is a time-consuming, expensive, arduous and often impossible task. Sewage testing is relatively inexpensive and can track accurately the movements up and down of COVID-19 prevalence in a community. A Parisian study [9] suggests that a population newly experiencing a rise in COVID-19 infections will exhibit that rise in excreted virus-containing stool about one week before showing visible symptoms of infection. Timely sewage testing may provide a One-Week (approximately) Early Warning System of population infection. While testing sewage is not the same as testing individuals, it is fast, inexpensive and informative to public decision makers.

### 1.2. Wastewater testing

Numerous sewage testing “proof-of-concept” projects have been recently reported. In addition to the aforementioned Parisian study [9], these include projects in Arizona [8], Montana [10], New Haven Conn. [11], Boston Massachusetts [12], Italy [13], the Netherlands [14] and many more. These reports demonstrate that the movements of viral loads in the sewage track well the movements up and down of known cases in the population, and often with a one-week early warning.

Analysis of wastewater in Italy has revealed the presence of SARS-CoV-2 in December, more than two months before known cases arose there [15]. Researchers at Italy’s National Institute of Health (ISS) reported that recent re-analysis revealed remnants of SARS-CoV-2 in the wastewater of Milan and Turin in December 2019. These findings point to the invaluable attribute of wastewater as an early warning system for impending SARS-CoV-2 community infection. Given such early warnings, tracing methods such as our Algorithm 2 could help identify regions within cities having the highest likelihood of serous outbreak. MIT spinoff Biobot claims to be “the first company in the world to commercialize data from sewage.” [16]. Biobot is now working with about 330 facilities in 40 states, returning analyses of sewage samples sent to them.

All of these activities are now evolving, with current test turn-around times disappointingly long, ranging from one to two weeks. Biobot claims to be headed for a 24-hour test in its Boston-area headquarters, which means perhaps a 3-day turnaround time total; that would get us closer to what is needed to have a true Early Warning System. There is another approach, building from tests successfully developed for other diseases in Africa, using only paper as the testing material to create what is being described as an instant “two-dollar test” [17]. A key member of the team of collaborating researchers is Dr. Zhugen Yang, a biomedical engineer at Cranfield University’s Water Science Institute in the U.K. He reports that the primary working system has been tested, but it needs some optimization and further validation. Ironically, the cause for the developmental delay is COVID-19, creating difficulty in accessing the lab, using sourcing reagents, etc.

With the current WTP analysis techniques, all we have to date is *community-averaged* predictions. There is no finer-grain resolution relating to the likely locations within the community of the rising virus.

### 1.3. Framing of our contribution

The focus on this paper is to use sampled sewage flows to gain new information about locations within the community of new COVID-19 infections. These samples are obtained from dynamically selected sewer system manholes. To the best of our knowledge this is the first paper that frames a procedure and develops an algorithm for sampling manholes to find the sources of the virus. While we develop algorithms for the solution, we do not view our major contribution as an algorithmic one.

Our manhole sampling procedures, to be successfully implemented in the field, will require a proven “Fast Test,” such as the one being created by Dr. Zhugen Yang. Since such a test not yet available, our algorithmic procedures are a bit ahead of the rest of the science of sewage system sampling. With so much current active research, we expect this gap to close quickly.

Any sewage system can be modeled as a tree network, with individual homes (single or multi-family), schools and businesses providing input flows into the system. Any sewage will flow in one given path from origin towards the single terminal node of the tree graph, the local WTP. Our research aims at finding a best or near-best sequence of manholes to sample and test in order to home in on the location of new infection(s) as quickly as possible.

There exists no standard literature review for this problem since knowledge of the possible existence of such a procedure did not previously exist. But the need is clear, and not only with COVID-19. There are numerous situations that require that we locate the source location for a substance in the sewage system originally detected at the WTP. For example, the firm, Advanced Sensor Technologies, offers sewage testing to locate the sources of illegal pH excursions in wastewater [18]. In discussing how they would use their sensors to identify the location of the offending address, they state,

“… These field portable installations could be moved further and further upstream away from the treatment plant until the excursions were detected and the location of the events could be narrowed to a manageable search area.” [18]

But “going upstream” does not define a unique path in the sewage pipeline network. The corporate literature continues,

“The point where the initial excursion was detected was followed into the branches until the grid area of the source could be narrowed down such that the number of facilities to be visited was minimized.” [18]

This can become a labor intensive, trial-and-error process. In a private communication to the authors, the company vice president says of our method, “This approach looks to be a very interesting route to isolating hot-spots.”

There are more potential applications of our work, for example, locating terrorist bomb makers, where bomb chemicals are detected either because they have been poured down the drain or ingested into the terrorist's blood stream and then excreted [19], and locating sources of illicit drugs and many other substances correlated with human health [20].

But our primary motivation has been Covid-19: zeroing in on the location of a newly infected individual. As we write this, many U.S. colleges and universities are monitoring manholes adjacent to student dormitories to detect the first signs of a new infection in the dormitory. Once such a signal is detected, then all the students in the dormitory are tested, and the infected one is removed and placed into supportive isolation. As one example, Professor Ian Pepper of the University of Arizona reports to us, “Here at the University of Arizona, the WEST Center is monitoring approximately 20 student dorms as part of our Campus Reentry Program designed to assure the health and safety of our students. Sampling of wastewater from the dorms will be accomplished via manholes with composite samplers.” Our work, when implemented with minimal labor and capital investment, will be able to provide the same or better level of granularity in identifying the newly infected person for an entire dispersed community, not only a concentrated student or adult residence hall.

We provide two algorithms. Algorithm 1 represents the case of a community known to have zero infections at time zero. Eventually, monitoring of the community’s sewage at its WTP indicates a new presence of COVID-19, likely from a “Patient Zero” within the community. Our algorithm dynamically selects a sequence of manholes to test for COVID-19, each selection dependent on the test results of the previous manholes. Eventually, with just a handful of manhole tests, we converge to Patient Zero’s neighborhood, usually to within 100 feet or so of his/her address. Algorithm 1 relies on a type of binary search (also similar to the bisection algorithm) that converges quickly to the neighborhood of the infected individuals(s). Algorithm 1 can be viewed as an application of “pooled sampling” [21], where the method focuses in on only a small fraction of potentially infected people, those being the only ones requiring individual tests for SARS-Cov-2. Without homing in on the infected neighborhood, only full community testing would find the infected person.

For Algorithm 2, there are assumed to be many infected individuals in the community. The aggregate WTP testing procedures previously described may be used to obtain community-averaged estimates on numbers of infections. Again, our concern is moving beyond the community average, to identify that neighborhood within the community that may be considered to be a “Hot Spot,” that is, an area having infection rates significantly above the community average. Such information is again useful to those who seek to do high-productivity testing to identify, treat, and isolate infected individuals. Algorithm 2 also uses a type of binary search, utilizes data from manhole testing of sewage, and sequentially selects the manholes to be tested. It stops once a presumed high-incidence neighborhood has been identified.

### 1.4. More on manholes and their testing

Each home in a community having a central sewer system has an underground pipe connecting the home’s wastewater plumbing to the under-street local sewer system. Using the descriptive adjectives of the Town of Surprise, Arizona [22], the building’s pipe to the property line is “the building sewer,” which then connects directly to the “lateral sewer,” which then connects to the community’s sewer pipe network. That network, a tree graph, has at least three types of pipes, each is ascending diameter for increased flows: branch sewer, main sewer and trunk sewer. The branch sewer, usually serving a limited number of buildings in a small geographic area, collects sewage from lateral sewers and conveys it to a main or trunk sewer. A main sewer collects sewage from two or more branch sewers acting as tributaries. The trunk sewer conveys sewage from many tributary main and branch sewers over large areas to the WTP.

The sewage, once in the community-owned system, flows in a unique path “downward,” leading to the WTP. A typical town will have hundreds of miles of under-street sewage pipes, the pipes accessible by manholes that are typically 100 to 500 feet apart [23]. As an example, the Town of Lexington, Massachusetts has 4,924 manholes distributed of its 171-mile sewer network, corresponding to about 183 feet between manholes [24]. The U.S. EPA estimates the number of sewer manholes nationwide to be about 12 million, averaging approximately 300 feet apart [25].

## 2. Methods

As discussed above, Algorithm 1 deals with a community with no known cases of COVID-19. Then, one day analysis of the inflow to the water treatment plant reveals the first presence of COVID-19, likely a single Patient Zero or household "Patients Zero." The goal is to act immediately to home in on the address of the infected person(s). Once the alarm is sounded, our action is to open a directed sequence of manholes, obtaining sewage fluid samples, testing them, and then extending our manhole testing, in a way that converges close to the address of Patient(s) Zero.

**Fig 1:**
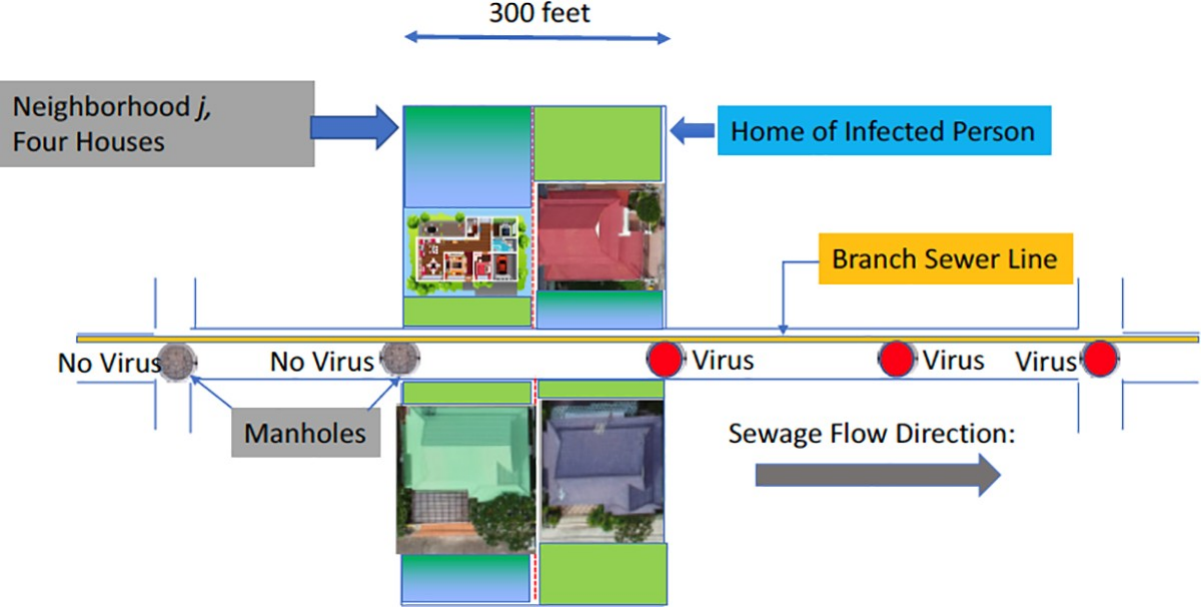
The small neighborhood catchment zone associated with a given manhole.

The home of Patient(s) Zero is likely to have two closest manholes, one “upstream” from the residence and the other “downstream.” See a typical neighborhood depiction in Fig 1. Any test of the upstream manhole, if it exists, would reveal no COVID-19 from the residence of the infected person(s). But the closest downstream manhole would provide that evidence. We seek to find that closest downstream manhole. If successful, we can then reduce our search for the infected person(s) to residences of only a few houses (i.e., all those houses first inputting to the same downstream manhole). By such targeted human testing, we may be able to stop any spread from Patient(s) Zero to the rest of the community. Our approach also extends in Algorithm 2 to cases where the virus has infected multiple residences in different neighborhoods. In such a scenario, the objective is the find the Hot Spot neighborhood with the highest number of infected homes.

In Algorithm 1, the single downstream manhole closest to the infected residence is called the ***source manhole*** or ***source node***. It is the only source of SARS-CoV-2 remnants entering the sewage tree network. This is the manhole we seek to identify. In Algorithm 2, there will be multiple source nodes, each being a closest downstream manhole to an infected neighborhood.

We start by assigning Bayesian probabilities [26] to all possible source manholes, each probability reflecting our initial belief that the infected address is assigned to that manhole. These probabilities reflect professional beliefs and need not be created by detailed data analysis, as the results are not that sensitive to their exact values. One could even use a simple heuristic, such as assigning each probability associated with a given manhole to be proportional to the number of residents in that manhole's “catchment” zone.

**Fig 2:**
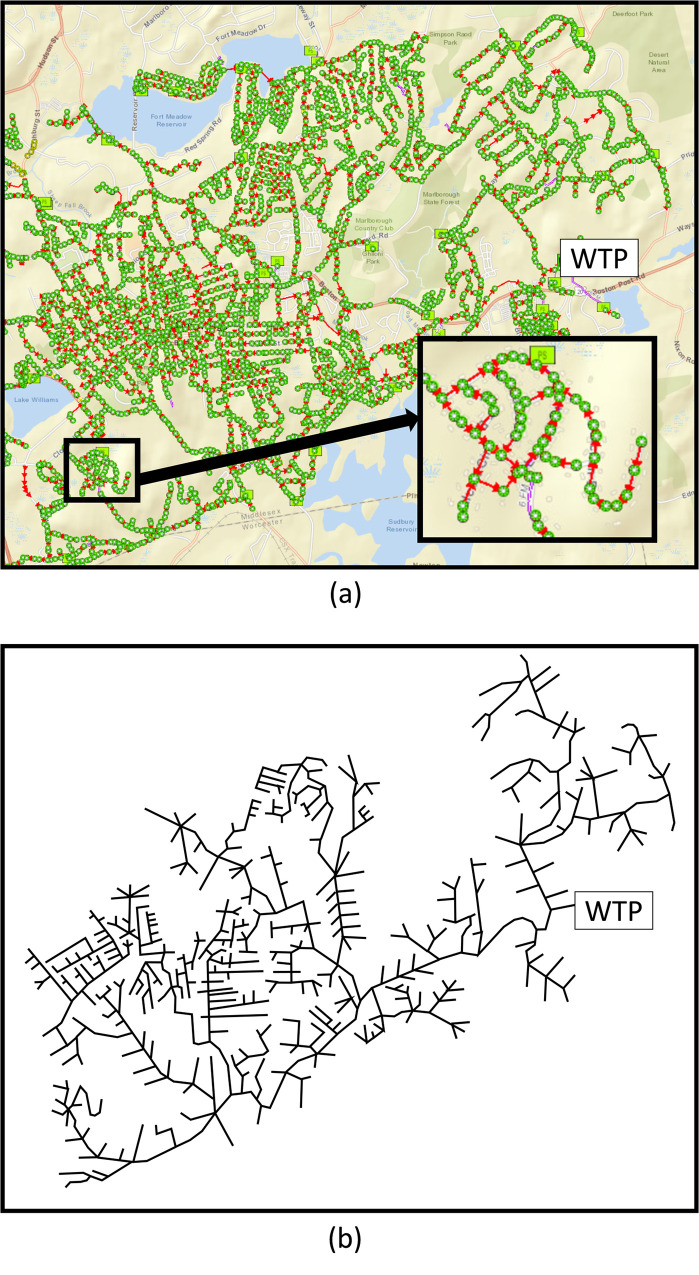
Top panel depicts a map of the Marlborough, Massachusetts wastewater removal system; the red arrows represent the direction of flow. The green circles are the manholes of the sewage pipe system. Bottom panel is a reduced network depiction of Marlborough’s sewage network. “WTP” in both panels represents the wastewater treatment plant.

Marlborough, Massachusetts is a typical New England small city, covering 22.2 square miles with a population of 38,500. After describing the logic of our algorithms first with small problems, we utilize a portion of the sewer system network of Marlborough to illustrate a realistic application of the algorithms. Fig 2 depicts a portion of the sewer pipe network for the city of Marlborough, which has one WTP and more than 3,000 manholes. In our model of the Marlborough system, we consider a subset of the system having a reduced 844 manholes, each a possible source node. Marlboro’s WTP, the terminal node of the tree network, is labeled in Fig 2. While a tree-network structure is not readily apparent by viewing the sewage system when superimposed on the town’s map (Fig 2(A)), we redraw its pipe network to reveal the tree structure as shown in Fig 2(B). We operate on the tree network depiction of Fig 2(B).

We now describe Algorithms 1 and 2 that find Patient Zero and the Hot Spot neighborhood respectively. We present two simple examples to illustrate the steps of each algorithm, each step representing the opening of a manhole and testing its contents. We then apply the algorithms on the reduced network of Marlborough, Massachusetts, and report the required number of manhole-opening steps to find the source(s) of infection in the city.

### 2.1. Algorithm 1: Finding Patient Zero

#### 2.1.1. Iteration 1

We use a variant of “binary search” [27, 28], an algorithm that finds the position of a target value within a sorted array. Binary search has the property that it discards about half of the remaining solution space at each iteration. We seek to do the same, to discard about half of the manholes at each iteration, none possibly being the desired source node. For each link we derive the “flow probability” as the probability that the infection is upstream of the link. *The flow probability of each link is the sum of Bayesian probabilities of source nodes upstream of that link*.

At Iteration 1 we seek a link in the tree that has about 50% of the Bayesian probabilities upstream from the link and the remaining Bayesian probabilities downstream from the link. Since it is unlikely that any subset of Bayesian probabilities will sum exactly to 0.5, we settle for the link whose probability is closest to 0.5. We identify a physical manhole within this link, open it, obtain a sewage sample, and then test it. If positive for SARS-CoV-2, then we know that the residence we are seeking and its associated closest downstream manhole are upstream from this point; as a consequence, we discard all network downstream nodes from this point. Else we know the reverse and discard all the upstream nodes.

#### 2.1.2. Iteration 2

We are left with a tree network that is a subgraph of the original network, now with fewer nodes than the previous iteration. We readjust the Bayesian probabilities on the surviving subgraph so that they sum to 1.0. We repeat the logic of Iteration 1: We find a link on this subgraph that has about half of the Bayesian probabilities upstream from the link and the remaining downstream. We identity a physical manhole on this “50–50” link, open it, obtain a sewage sample, and then test it. This is Test #2. If positive for SARS-CoV-2, then we know that the residence we are seeking and its associated closest downstream manhole are upstream from this point; as a consequence, we discard all downstream nodes from this point. Else we know the reverse and discard all the upstream nodes. The above process continues until we have found the manhole of Patient(s) Zero.

**Fig 3:**
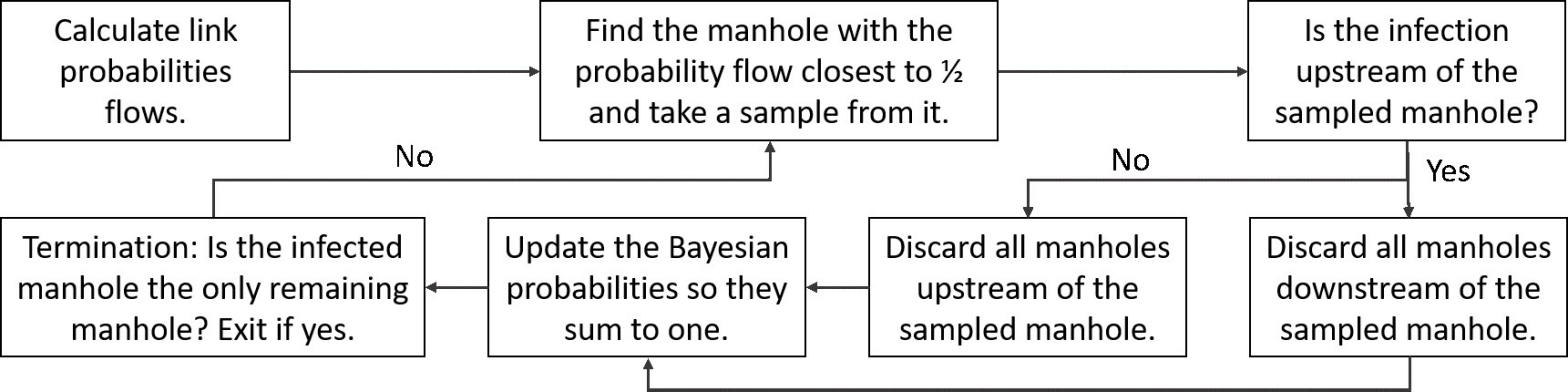
Algorithm 1 flowchart.

In summary, Algorithm 1 is detailed in the flowchart of Fig 3. As the number of remaining manholes decreases in each iteration, it is obvious that that algorithm always converges to the infected manhole.

**Fig 4:**
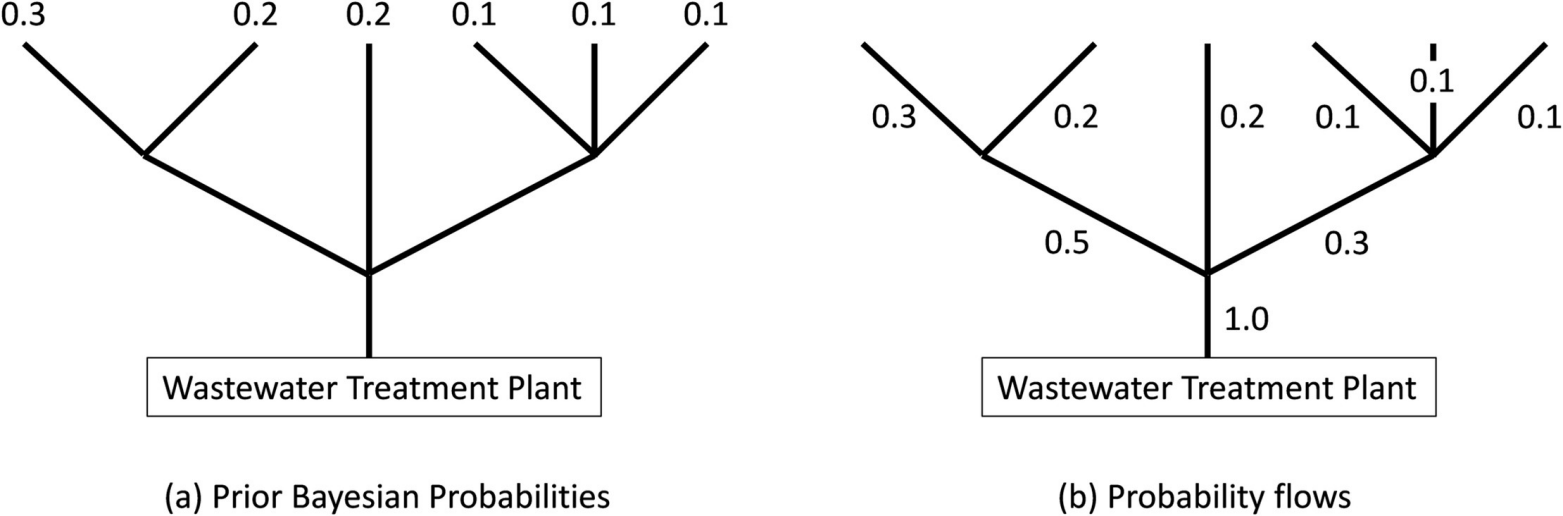
Bayesian probabilities and probability flows of a simple network.

**Fig 5:**
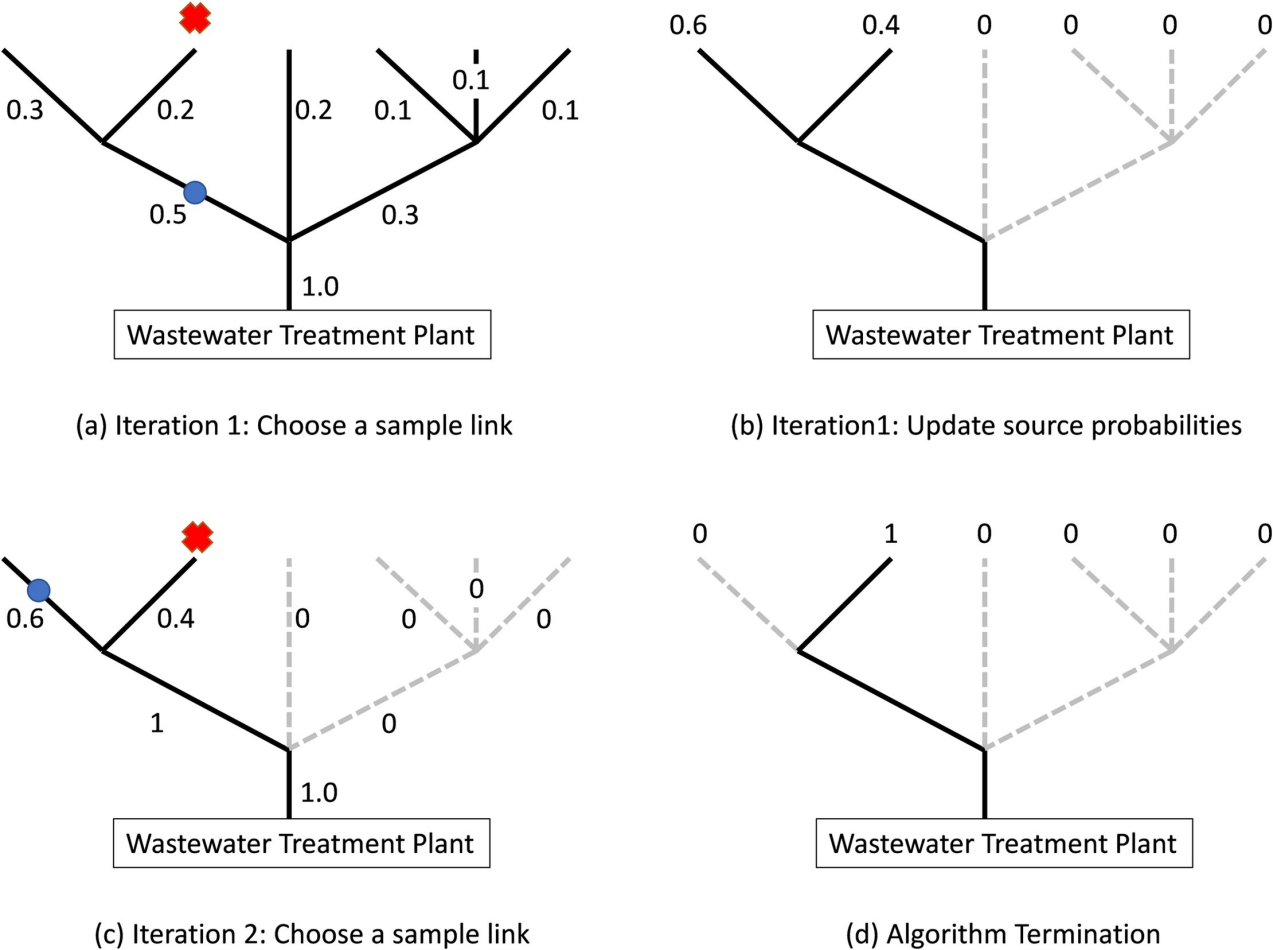
Bayesian probabilities and associated probability flows of a simple network. The infected node is depicted with a red “X” and the sample link is depicted with a blue circle. The dashed lines depict eliminated links.

We illustrate the steps of Algorithm 1 with a simple example of Figs 4 and 5. As mentioned above, each source node is assigned a Bayesian probability as shown in Fig 4(A). For each link of the tree we now assign a corresponding probability flow as shown in Fig 4(B). Our algorithm sequentially samples a set of links using Bayesian probability flows as a measure of the virus intensity in each link. Note that the sum of probability flows into the WTP is one.

The infected node (with an unknown location) is depicted with a red “X”. In Iteration 1 we choose to sample the link depicted with a blue circle in Fig 5(A) since it has probability flow of 0.5 and all other probability flows are less than 0.5. Because the sample tells us that the infection is upstream of the left link, we can discard the central and right links (Fig 5(B)). In Iteration 2 we normalize the probabilities (Fig 5(B)), recalculate the probability flows. Now we have a tie in the proximity to 0.5, and we choose to sample the link with the larger flow of 0.6, as shown in Fig 5(C). Because the sample tells us that the infection is downstream of the left link, we discard the left-most link. We can now terminate the search since the infected link is identified (Fig 5(D)).

### 2.2. Algorithm 2: Finding the Hot Spot neighborhood

In Algorithm 2 we consider two or more nearby nodes as comprising a neighborhood, the exact definition is left to the operator of the algorithm. As a preliminary step in Algorithm 2, we first sample and record the virus load in the intake sewage flow at the stem link of the tree, the single link that connects the tree network to the WTP. This sample gives us the overall total virus load in the city. Our algorithm seeks to find the neighborhood with the largest contribution to total system viral load.

#### 2.2.1. Iteration 1

We use again a type of binary search to reduce the network until we are left with the Hot Spot neighborhood. We first record the total Coronavirus in-flow *F* entering the wastewater treatment plant and multiply all node probabilities and link probability flows by *F*. Given the Bayesian probabilities and total virus load *F*, the revised flow *f* on any given link is now the *expected* virus load on that link. The actual viral flows will differ, as to be revealed by sampling. We then sample the branch having expected virus flows upstream and downstream closest to a virus flow of *F*/2. We identify this link and obtain a sewage sample. This step is the same as Step 1 in Algorithm 1. If the recorded network viral load at the sampled link from upstream exceeds *F*/2, we discard the downstream nodes. Else we know the reverse and discard all the upstream nodes.

#### 2.2.2. Iteration 2

We now have a tree network that is a subgraph of the original network, usually with significantly fewer nodes. We re-normalize the Bayesian probabilities on the subgraph and repeat the logic of Iteration 1 to open and test a physical manhole on a new “50–50” link. We discard upstream or downstream nodes of the tree in the same way as Iteration 1. The process continues until we are within a reasonable vicinity of the Hot Spot neighborhood. The stopping rule is human-based, subjective, depending on the size of the neighborhood and other considerations. By the viral-load-comparison mechanism of selection, we see that at each iteration the surviving subgraph has a higher average level of viral load per node than in any previous and now discarded subgraph. We can view the logic as a steepest ascent heuristic, always obtaining a subgraph with higher average viral load. Being a steepest ascent algorithm, we are not guaranteed to find the hottest “Hot Spot,” as there may exist a small neighborhood in a now-discarded subgraph having more viral intensity than that which we find with the algorithm.

In summary, Algorithm 2 follows the steps in the flowchart of Fig 3 but has a different termination criteria. We adopt a stopping rule that terminates the algorithm if the detected viral load divided by the number of upstream source nodes from two consecutive samples is less than a predefined threshold. (In practice, we believe that the stopping rule would be enacted by humans, using subjective decisions.)

**Fig 6:**
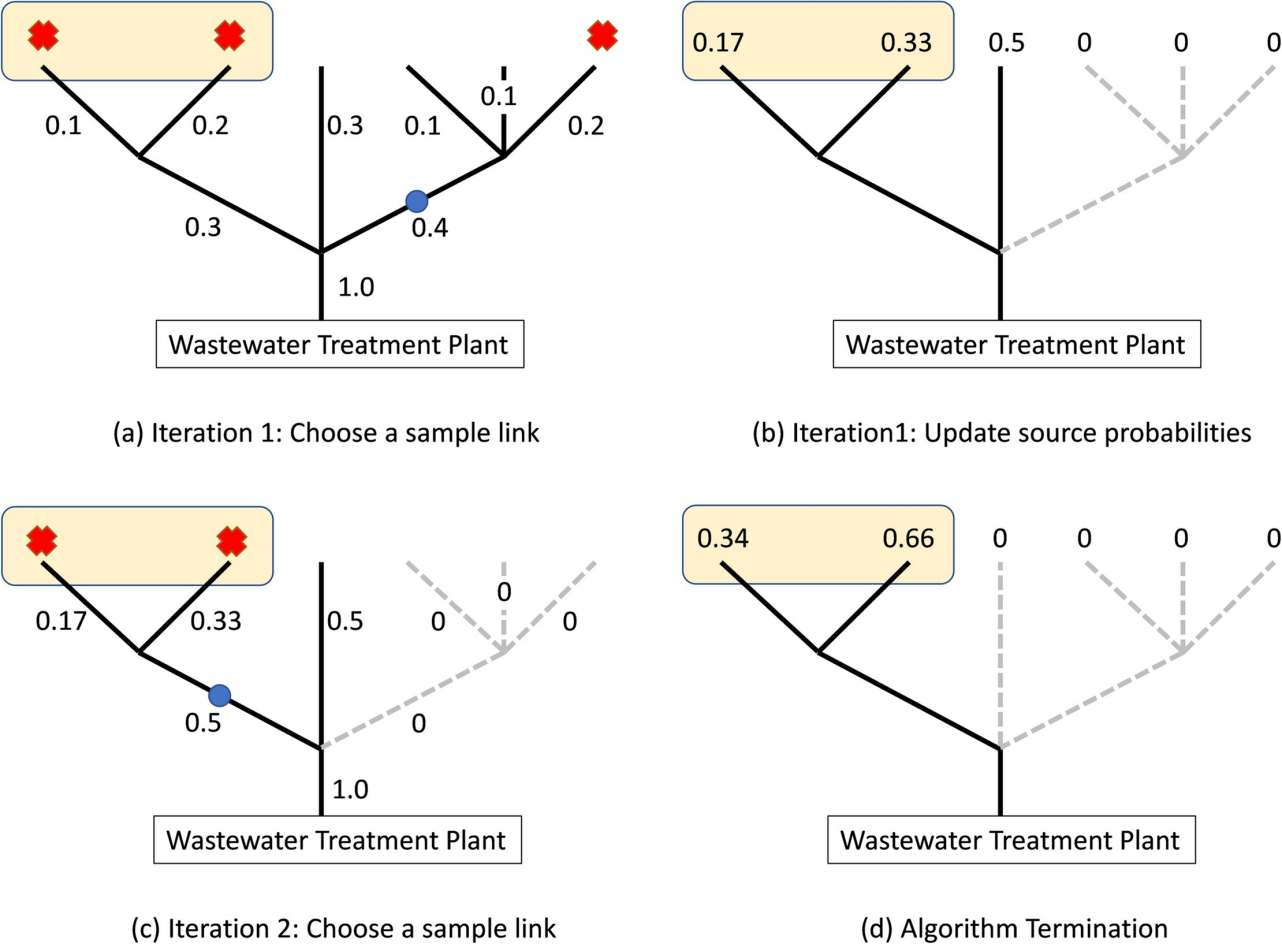
Bayesian probabilities and probability flows of a simple network. The infected nodes are depicted with a red “X” and the sample link is depicted with a blue circle. The dashed lines depict discarded links. The yellow box depicts the Hot Spot neighborhood.

We illustrate the steps of Algorithm 2 with a simple example that has three infected nodes as shown in Fig 6(A). The total level of infection at the stem link is *F* = 1. The infected nodes (which are not known to the algorithm) are depicted with a red “X”. In Iteration 1 we choose to sample the link depicted with a blue circle in Fig 6(A) since it has probability flow 0.4 (closest to 0.5) and all other flows are less than 0.4. We discard the right branch because the sample detected only 1/3 of the total recorded viral load, *F* = 1 (Fig 6(B)). In Iteration 2 we re-normalize the probabilities (Fig 6(B)) and choose to sample the left branch which has a probability flow of 0.5. This sample tells us that 2/3 of the stem link infection is from the upstream nodes. As there are only two nodes upstream of the sample point, we terminate the algorithm as we have found the Hot Spot neighborhood (Fig 6(D)).

### 2.3. Performance analysis of Algorithm 1

We assess the performance of Algorithm 1 by comparing it to a standard bisection algorithm [27, 28] which has a similar functionality of discarding a set of manholes at each iteration. In the bisection algorithm, we choose to sample a manhole that has (approximately) an equal number of manholes in its upstream and downstream. The algorithm discards half of the nodes at each iteration until it finds the infected manhole.

**Fig 7:**
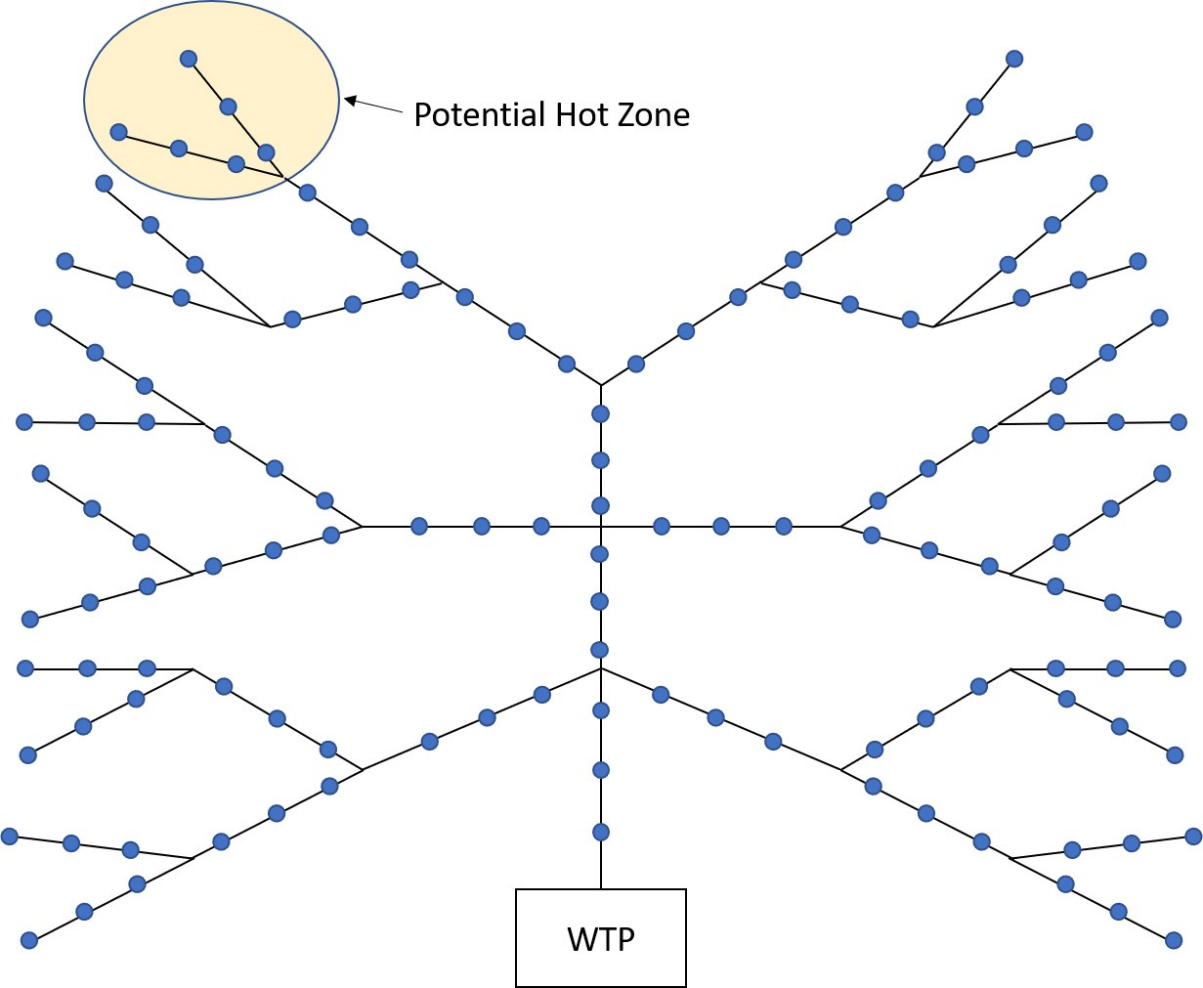
A stylized network.

Although the bisection algorithm ignores the Bayesian probabilities, it benefits from reducing the search space effectively always by half at each iteration. We compare Algorithm 1 to the bisection algorithm by applying them both to the stylized network of Fig 7. The network has the same number of manholes on each link (three manholes in the depiction of Fig 7). By increasing the number of manholes on each link, we can control the size of the network.

**Fig 8:**
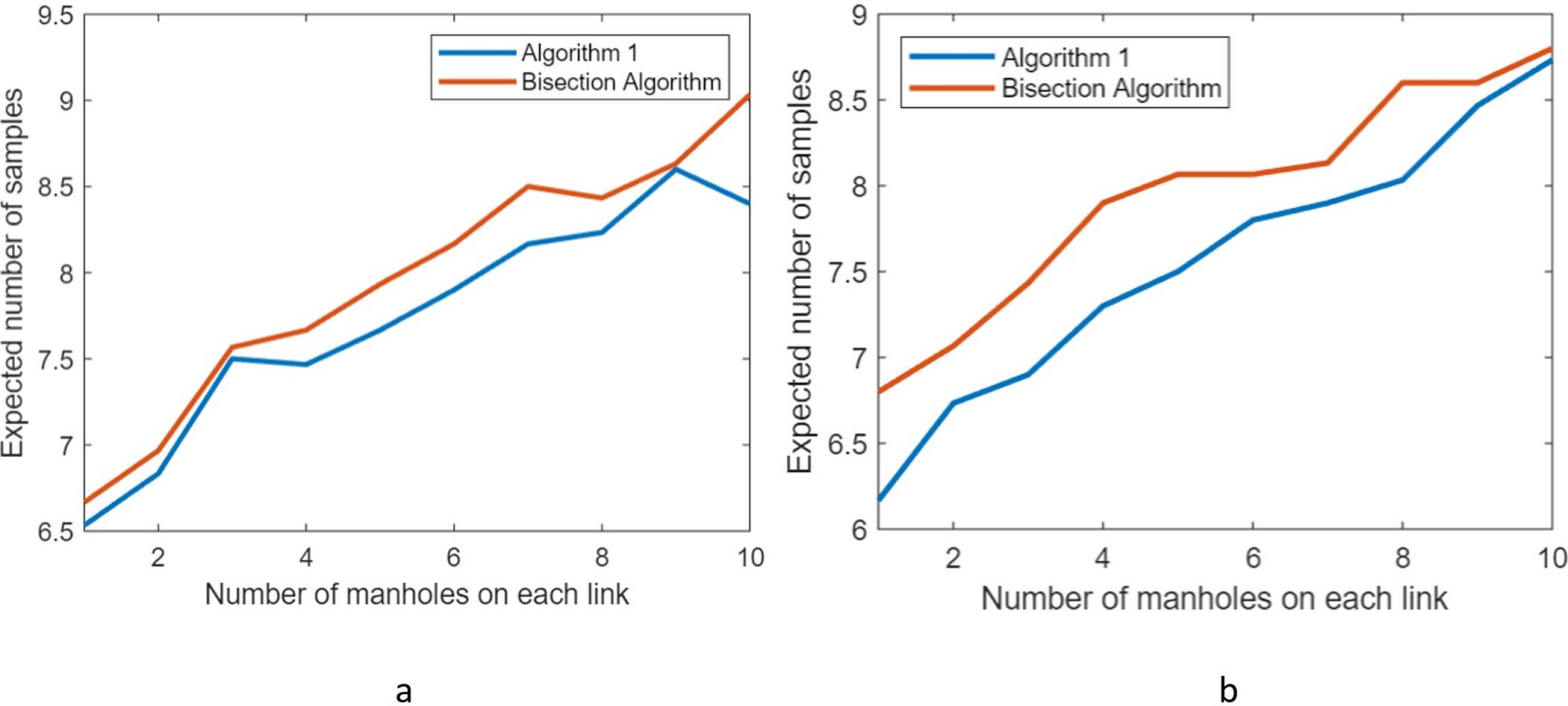
Mean of the number of samples when (a) the Bayesian probabilities are drawn from a unit uniform distribution, and (b) there is a 20% probability that the infection is in the potential Hot Zone.

We perform two sets of tests on the network. We first assume that the Bayesian probabilities are randomly generated from a unit uniform distribution and normalize the probabilities, so they sum to one. We generate varying network sizes by increasing the number of manholes on each link. For each network size, we select via Monte Carlo sampling the infected manhole 30 times and report the mean of the number of samples in Fig 8(A). According to the results, Algorithm 1 always outperforms the bisection algorithm in terms of the expected number of samples. The maximum and minimum percentage difference in the expected number of samples is 5% and 0%, respectively.

We next generate the Bayesian probabilities with the condition that there is a 20% probability that the infection is in the potential Hot Zone of Fig 8. For each network size, we select via Monte Carlo sampling the infected manhole 30 times and report the mean of the number of samples in Fig 8(B). According to the results, Algorithm 1 always outperforms the bisection algorithm and has a maximum percentage difference of 11%. According to Fig 8, it is evident that Algorithm 1 increases its dominance over the bisection algorithm when some manholes have larger Bayesian probabilities of being infected.

### 2.4. Returning to Marlborough, Massachusetts

**Fig 9:**
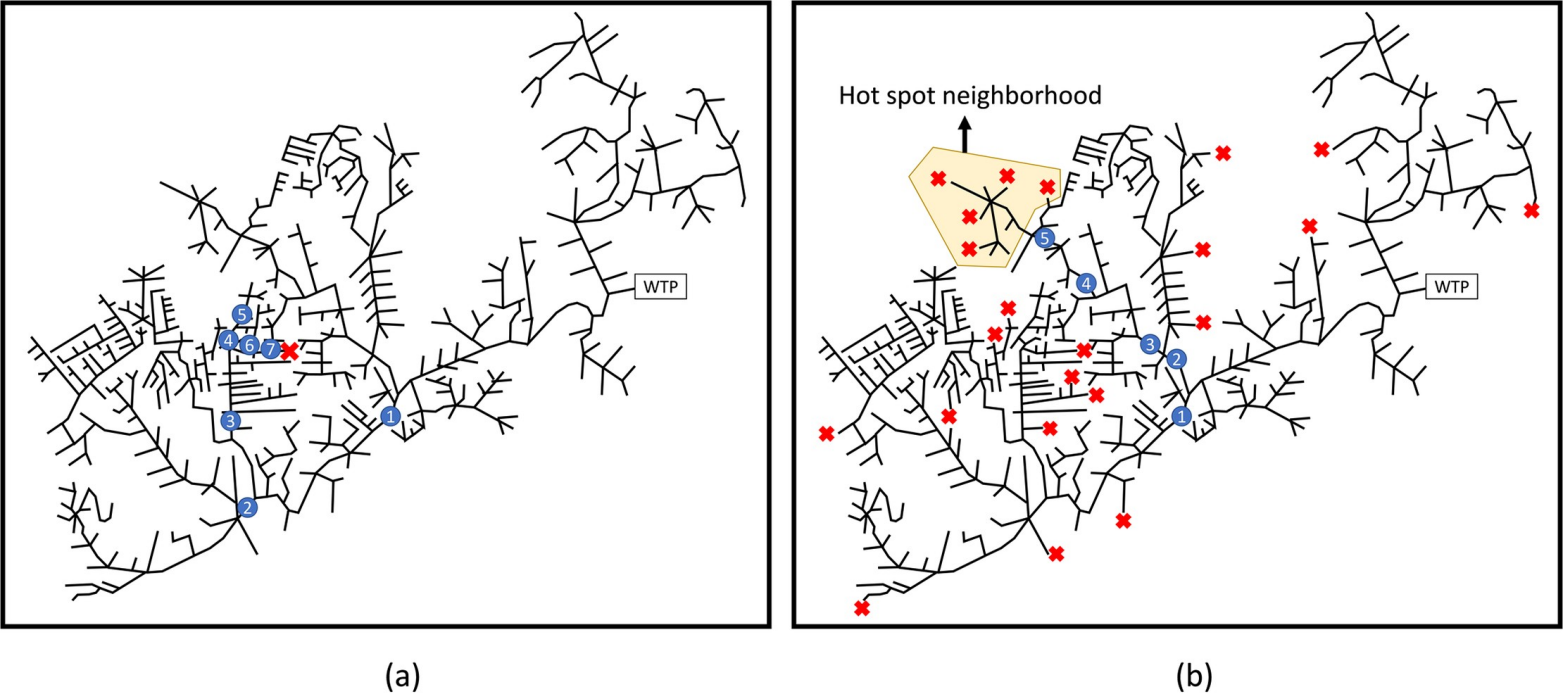
Left and right panels depict the Patient Zero and the Hot Spot neighborhood scenarios, respectively. In the right panel, each red X is an infected manhole and the Hot Spot neighborhood is depicted in yellow.

We now implement our two algorithms on our reduced model of the Marlborough sewage network. To generate the nodal Bayesian probabilities, we independently draw random numbers from a unit uniform probability distribution for each potential source manhole and normalize the random draws so that the Bayesian probabilities sum to one. We first consider the Patient Zero scenario where only a single residence is infected, as shown by the red X near the city center in Fig 9(A). In practice, municipal data sources can provide more accurate estimations of these Bayesian probabilities.

As shown in Fig 9(A), Algorithm 1 required only 7 samples to locate the infected source node. Iteration 1 divided the network into two relatively equal size subgraphs. Iterations 2 and 3 helped us to determine the general whereabouts of the infected zone. Iterations 4, 5, 6, 7 pinpointed the exact location of the infection.

Switching to Algorithm 2, we next consider the Hot Spot neighborhood scenario where several nodes are infected as shown in Fig 9(B). We have randomly generated the locations of infected nodes by taking random draws from the Bayesian probability distribution. As shown in Fig 9(B), the algorithm converged in five samples to the neighborhood having the largest number of infected nodes.

**Fig 10:**
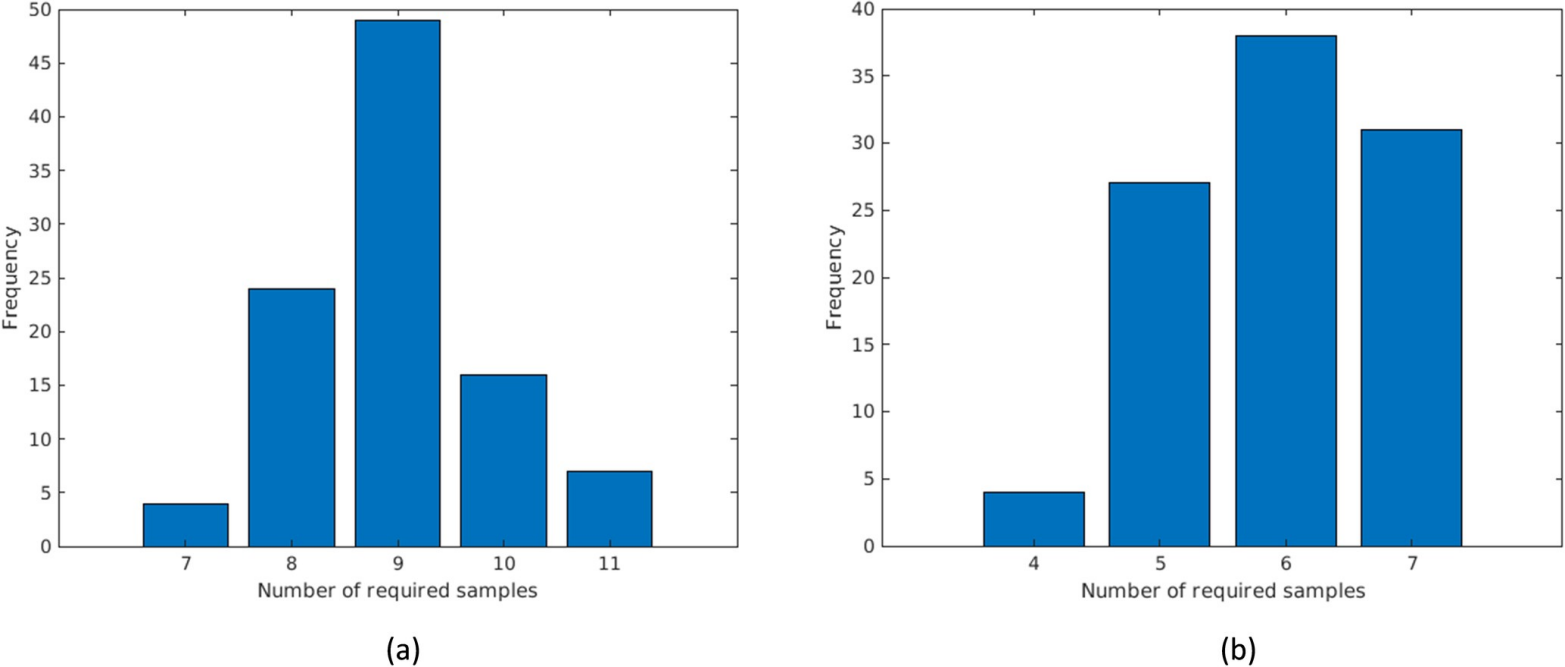
The number of sampled manholes to find (a) Patient Zero, and (b) the Hot Spot neighborhood in a reduced Marlborough network.

We now discuss the performance of the two algorithms for Marlborough, assuming widely different locations of infection. Our previous analysis shown in Fig 9(A) depicted a single scenario of the problem. Subsequently, we randomly generated 100 scenarios, each having a new set of Bayesian probabilities. For each scenario, the infected node is randomly selected by taking a Monte Carlo random draw from the Bayesian probability distribution. For each scenario, we then use Algorithm 1 to find the infected node. The frequency distribution of the number of required samples is shown in Fig 10(A), ranging from a minimum of 7 to a maximum of 11 samples being required over 100 generated scenarios. This is a surprisingly small number of samples considering the size of the network with its 844 manholes. We note our use of Bayesian probabilities allows us to find the infected manhole is as little as 7 manhole samples.

We use a similar approach to generate scenarios for the case of finding the Hot Spot neighborhood. Recall that each source manhole has an assigned Bayesian probability. For each scenario, we use Monte Carlo Sampling of the Bayesian probabilities to determine whether or not any given node is infected, iterating over all nodes. We generated 100 different scenarios. In Fig 10(B), we present the number of samples to find the Hot Spot neighborhood, showing a minimum of 4 and maximum of 7 samples. Algorithm 2 requires fewer samples because it does have to zero in on a single infected node, instead it finds the vicinity of the Hot Spot neighborhood.

**Fig 11:**
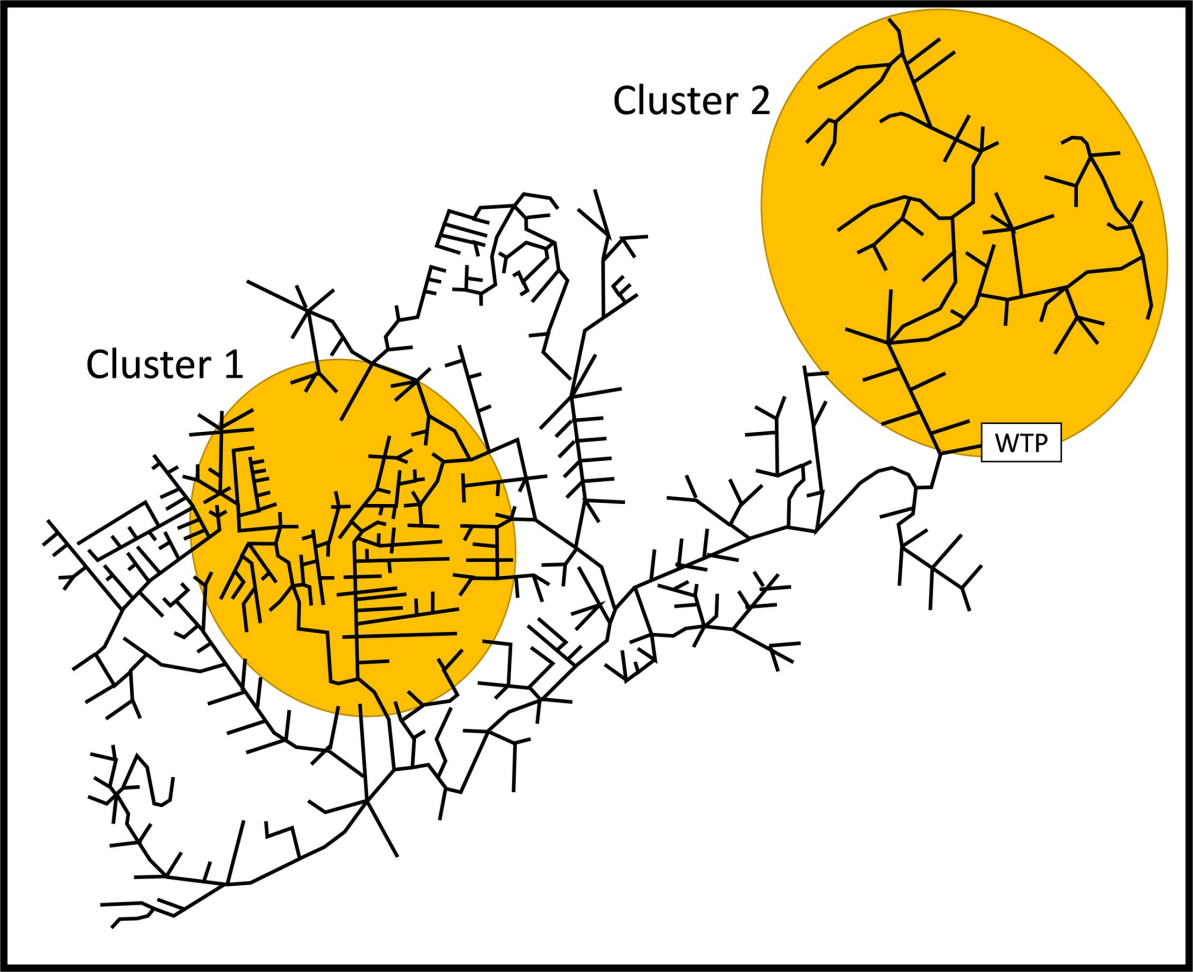
Marlborough, Massachusetts, with two infection clusters. Sum of source node Bayesian probabilities in Clusters and 1 and 2 is 0.5 and 0.25, respectively. For the remainder of the city, the sum of source node probabilities is 0.25.

We now consider a scenario where we have prior information that two geographical areas of Marlborough have a higher chance of being infected. We consider two ***clusters*** as likely Hot Spots shown in Fig 11. The sum of Bayesian probabilities of the source nodes in Clusters 1 and 2 is 0.5 and 0.25, respectively. The sum of Bayesian probabilities over the rest of the city is 0.25. We generate the Bayesian probabilities in the same way as above but ensure that the sum of probabilities in Cluster 1 is 0.5, Cluster 2: 0.25, and the remainder of the city: 0.25. Similar to above, we randomly generate 100 scenarios, each having a new set of Bayesian probabilities, and use Monte Carlo sampling of the Bayesian probabilities to choose the infected nodes.

**Fig 12:**
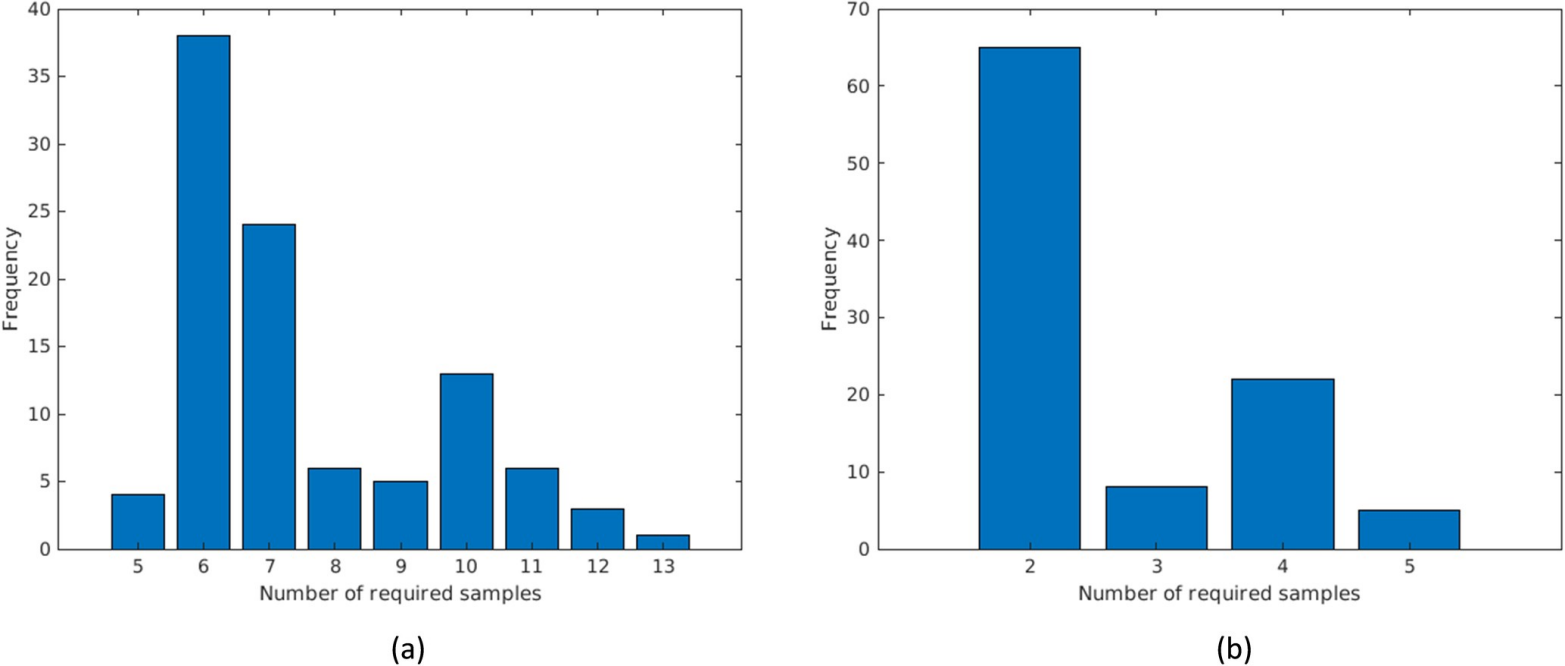
The number of sampled manholes to find (a) Patient Zero, and (b) the Hot Spot neighborhood in a reduced Marlborough network when there are clustered infections.

We present the number of required samples for the two algorithms in Fig 12. Algorithm 1 in Fig 12(A) has two peaks in the distribution of the required number of samples. The left peak is associated with Cluster 2 which has fewer source nodes and is less intricate to search than Cluster 1. It is easier to find the infected node in Cluster 2. The right peak is associated with Cluster 1 which typically required more samples to find Patient(s) Zero. Algorithm 2 in Fig 12(B) also has two peaks for the two clusters.

We note that the maximum number of samples of Algorithm 1 with clusters is 13 (Fig 12(A)), in contrast to 11, its counterpart without clusters (Fig 10(A)). The cluster scenario may require more samples than the no-cluster scenario in cases when the infected node (or the infected neighborhood in the Hot Spot case) is outside of the clusters. In such cases, one might say that the Bayesian probabilities give misleading information on the whereabouts of the infection, thus, more samples are required.

We compare the two algorithms in the cluster and no-cluster scenarios in Table 1. The mean number of samples is smaller in the cluster compared to the no-cluster scenario. In Algorithms 1 and 2, the cluster scenario reduces the mean number of samples by 14% and 22%, respectively. However, the cluster scenario has a larger variance in the number of samples, which, as explained above, is predominantly due to cases where the infected nodes are outside of the clusters. According to Table 1, we have a larger right-tailed skew in the number of samples when the infection is clustered, showing that we can find the Hot Spots with a fewer number of samples compared to the no-cluster scenario.

**Table 1:** Mean, variance, and skewness of the number of samples required to find Patient Zero or the Hot Spot neighborhood with and without infection clusters.

	Mean	Variance	Skewness
Random Infections: Patient Zero	8.98	0.75	0.42
Clustered infections: Patient Zero	7.71	3.39	1.09
Random Infections: Hot Spot	5.96	0.75	-0.31
Clustered Infections: Hot Spot	4.66	1.48	1.42

Finally, moving away from clusters, the two algorithms work well in large as well as small networks. As an example, in a test of Algorithm 1 on randomly generated networks with approximately 3,000 links and 1,000 source nodes, we found typically that only 11 manhole samples were required to home in on the desired location. Applying to both simulated and actual sewer networks, we perform extensive computational and complexity analysis in [29].

## 3. Reflections

We have presented two algorithms that utilize viral markers of SARS-CoV-2 contained in human stool, and thus also present in human sewage, to help identify location(s) in a community of individuals currently infected. Each algorithm recommends a dynamically-dependent sequence of manholes to sample in order to home in on the locations(s) of the infected individuals. In the first case, we are seeking one or a small number of persons with COVID-19 living at the same address. In the second we are seeking a Hot Spot neighborhood within the community.

The programmed algorithms are very fast on any computer, and they suggest only a small fraction of a community’s manholes need to be sampled and tested. For Algorithm 1, for instance, one could double the size of the community being analyzed and typically only add one extra manhole to sample and test! This important property derives from the binary search logic that attempts to cut away half of the eligible manholes at each iteration.

As mentioned earlier, our work is a bit ahead of the current science and technology of COVID-19 tracing within a community. There are the major issues ahead of us, for our algorithmic approach to work well in practice.

### 3.1. Fast testing

For our methods to work, we need rapid testing on the spot at the manholes, each test administered to the newly obtained sewage sample. There are current efforts to create such tests, including the previously cited “fast two-dollar test.”

### 3.2. Sewage system realities

Municipal sewage systems are not pristine like hospital or university labs. They are notoriously under-maintained. Foreign substances other than human waste can occupy these systems. Thus, it remains to be seen how well these systems can accurately maintain and convey virus remnants during the winding tributary-and-main-line journey to the sewage treatment plant.

We have verified that manhole sampling is feasible and reasonably priced. In Lexington, Massachusetts, to test four manholes, gathering up to one liter of fluid at each location, takes at most four hours of a two-person crew, from start to finish. Quoted cost: $350. If either of our algorithms should find a sequence of say eight or fewer manholes to test, such tests could easily be carried out in one day, assuming there is a fast, on-site test for COVID-19 that could be carried out by the crew. The “two-dollar” paper test being developed by Dr. Zhugen Yang, if successful, would be perfect for this purpose.

### 3.3. Lack of time averaging

Most of the recent sewage content analysis by researchers cited in the opening of this paper uses time-averaged measurements of SARS-CoV-2 remnants. We do not have the luxury of time averaging with our fast testing and quick identification of the next manhole to test. This should not be a problem for Algorithm 2, since it focuses on a populated neighborhood of infected individuals and does not seek to estimate full viral load, only relative viral load in contrast to other neighborhoods. But it can be a problem with Algorithm 1 since we are dealing there with one or a small number of individuals. The viral signature they input to the sewage system at 9:00 AM is likely to be a burst of viral load, perhaps then followed by no input until later in the day or perhaps even the next day. So, there is a reasonable chance that this individual’s viral signature in the sewage flow varies up and down during the day, possibly causing our sequential sampling system to miss the signature. The science of fluid dynamics suggests that the initial burst should dissipate and spread out over the journey through the system, thus smoothing the signature in the sewage flow. Such smoothing, if it occurs, could help our analysis. Also, many people having COVID-19 also have persistent diarrhea, and that unfortunate condition would tend to even the signature flows over the course of a day. Finally, in private conversations with a senior hydraulic engineering research professor, we learn that no real-time sewage flow is required in our testing to reveal remnants of COVID-19. With no current flow, we sample and test the sludge in the manhole to verify (or not verify) upstream presence of the virus. Operationally, we see many question marks going forward. Only future in-the-field research will help clarify these nontrivial issues.

### 3.4. Privacy

The focus of each algorithm is to speed testing of residents in a community by identifying “most likely” addresses of infection. Algorithm 1 homes in on a few houses on one street, while Algorithm 2 focuses on identifying a likely neighborhood, say comprising several city or town blocks. Residents who are subsequently selected for testing by our focused new means may object on privacy worries. In the context COVID-19, privacy concerns have been expressed over use of cell phone tracking, in-house monitoring devices, facial recognition software and more [30]. It is too early to know if tracking of virus-laden sewage would spark similar concerns.

### 3.5. More methodological research needed

This is the first paper to offer a way to test manholes sequentially to home in on locations of individuals having COVID-19.

We did not devote attention to evaluating the relative Bayesian probabilities. Their careful estimation for this procedure could be an entirely separate paper, and in practice, could result in much-improved performance. For instance, a neighborhood in which the majority of people have jobs that require leaving the house and working in an environment with substantial human interaction is likely to generate more COVID-19 cases than one in which most residents can work from home via the Internet. These differences can be expressed by markedly different values of the Bayesian probabilities assigned to neighborhoods.

Once our algorithmic procedures are tried in practice, building a database of results along with practical problems, there undoubtedly will be a need to fine tune the algorithms and related procedures to adapt to the realities in the field. We note that that nucleic acids degradation rate may introduce some bias in the results of the algorithm which should be considered in future research.

## Supporting information

S1 File (M)

S2 File (M)

S3 File (M)

S4 File (M)

S5 File (M)

S6 File (M)
